# Characterization of dermatan sulfate and chondroitin sulfate profiles during brain regeneration in *Styela plicata*

**DOI:** 10.1111/febs.70598

**Published:** 2026-05-21

**Authors:** Taynan Motta Portal, Samara de Oliveira Pimentel, Bruno da Costa Rodrigues, Mauro Sérgio Gonçalves Pavão, Rodrigo Nunes da Fonseca, Cintia Monteiro de Barros

**Affiliations:** ^1^ Laboratório Integrado de Biociências Translacionais, Instituto de Biodiversidade e Sustentabilidade – NUPEM Universidade Federal do Rio de Janeiro (UFRJ) Brazil; ^2^ Laboratório Integrado de Ciências Morfofuncionais, Instituto de Biodiversidade e Sustentabilidade – NUPEM Universidade Federal do Rio de Janeiro (UFRJ) Brazil; ^3^ Laboratório do Tecido Conjuntivo, Instituto de Bioquímica Médica (IBqM) Universidade Federal do Rio de Janeiro (UFRJ), Hospital Universitário Clementino Fraga Filho Brazil

**Keywords:** carbohydrate sulfotransferase, dermatan sulfate epimerase, glycosaminoglycans, neuroregeneration, tunicate

## Abstract

Neuroregeneration is the ability of nervous tissue to renew itself after injury. This process is highly limited in mammals. In contrast, marine chordates such as ascidians display a remarkable regenerative capacity, making them valuable models to understand neural regeneration. This study investigated dermatan sulfate (DS) and chondroitin sulfate (CS) profiles during brain regeneration in the ascidian *Styela plicata*. Neurodegeneration was induced by 3‐acetylpyridine (3‐AP), after which neural complex (NC) analyses were conducted using histology, RT‐qPCR, liquid chromatography, behavioral testing, and phylogenetic methods at 1,5 and 10‐day postinjection. One day after treatment, the cerebral ganglion exhibited significant degeneration, followed by morphological and molecular recovery at 10 days, when neuronal and synaptic markers returned to control levels. Gene expression analyses revealed early upregulation of C‐6, C‐4, and C‐2 sulfotransferases in the final stage. For the first time, the presence of dermatan 2,6 sulfate (D2,6S) and chondroitin 4 sulfate (C4S) in the cortex of the cerebral ganglion was described and 2,6 sulfate disaccharides were found to make up the majority of the NC, emphasizing their relationship with regeneration. A behavioral test revealed that co‐injecting 3‐AP and D2,6S restored the compromised siphon movements. Finally, ascidian DS epimerase (DSE) was most closely related to vertebrate DSE type 2, which is found in the brains of mammals. Together, these results indicate that specific glycosaminoglycan sulfation patterns are dynamically regulated during neural regeneration in ascidians. This study provides new insights into the molecules and strategies promoting neuroregeneration in vertebrates and advances the field of regenerative medicine.

## Graphical abstract

Adult ascidians are capable of regenerating their entire brains following injury, and glycosaminoglycans have been shown to either promote or inhibit neuritogenesis. In this study, dermatan 2,6‐disulfate (D2,6S) was identified as the predominant complex carbohydrate in the ascidian brain. Both the expression of its biosynthetic enzymes and the D2,6S content increased after injury. These findings suggest that D2,6S plays a role in neural recovery.

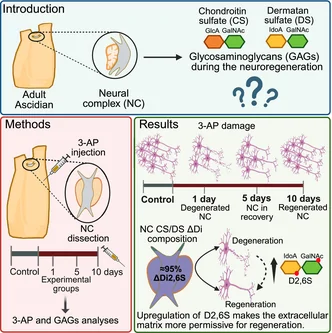

**Abbreviations**
3‐AP: 3‐acetylpyridine
C4S: chondroitin 4 sulfate
C4ST: chondroitin 4‐O‐sulfotransferase
C6ST: chondroitin 6‐O‐sulfotransferase
CNS: central nervous system
CS: chondroitin sulfate
D2,6S: dermatan 2,6 sulfate
DS: dermatan sulfate
DSE 1: dermatan sulfate epimerase type 1
DSE 2: dermatan sulfate epimerase type 2
DSE: dermatan sulfate epimerase
ECM: extracellular matrix
GAGs: glycosaminoglycans
GalNAc: N‐acetylgalactosamine
GlcA: glucuronic acid
HEP: heparin
HS: heparan sulfate
IdoA: iduronic acid
L: like
NC: neural complex
SYN: synaptophysin
TUJ: β‐III‐tubulin
UST: 2‐O‐sulfotransferase

## Introduction

Regeneration is the ability of tissues to renew and rebuild themselves following damage [1, 2]. The regenerative process is a phenomenon that has long captured the scientific community's interest, especially with regard to the regeneration of the central nervous system (CNS). Regenerative capacity varies by phylum and is not uniformly distributed across organisms or tissues throughout their lifespan. The brain is considered one of the organs with the most limited plasticity and renewal capacity, particularly in adult mammals [3, 4].

CNS regeneration is a complex process involving various cellular and molecular mechanisms, including neurogenesis, neuritogenesis, axonal guidance, and synaptogenesis [5, 6]. Macromolecules, including proteins and complex carbohydrates called glycosaminoglycans (GAGs), which are key components of the extracellular matrix (ECM), play a significant role in these mechanisms and have attracted attention due to their neural bioactive properties [7, 8, 9, 10]. GAGs influence cell‐to‐cell and matrix‐to‐cell regulation and can modulate signaling pathways and growth factors within nervous tissue [11, 12]. These long, linear, sulfated carbohydrates are classified according to their structure into six types: hyaluronic acid (HA), keratan sulfate (KS), heparin (HEP), heparan sulfate (HS), chondroitin sulfate (CS), and dermatan sulfate (DS) [13].

CS is composed of glucuronic acid (GlcA) and N‐acetylgalactosamine (GalNAc), whereas DS is formed through the epimerization of GlcA into iduronic acid (IdoA) via the action of DSE [14]. Despite this difference, CS and DS are structurally analogous. Vertebrates possess two types of DSEs: DSE type 1 (DSE 1) and DSE type 2 (DSE 2). These are encoded by the *dse* and *dsel* genes, respectively [15]. Five types of sulfotransferases, including chondroitin 4‐O‐sulfotransferase (C4ST), dermatan 4‐O‐sulfotransferase, chondroitin 6‐O‐sulfotransferase (C6ST), GalNAc‐4‐sulfate 6‐O‐sulfotransferase (GalNAc4S‐6ST), and 2‐O‐sulfotransferase (UST), are responsible for adding sulfate groups to GalNAc, IdoA, and GlucA at specific positions [16, 17]. These enzymes act differently depending on the tissue, contributing to the high structural variability of CS and DS and generating varying degrees of sulfation [17].

Different sulfation patterns are associated with distinct GAG functions. C4S has been shown to inhibit axonal cone growth and block nervous regeneration. In contrast, D2,6S has been found in mammalian neurogenic sites, where it promotes neuritogenesis and cellular differentiation, supporting nervous regeneration [8, 10, 18, 19, 20, 21, 22].

In contrast to mammals, ascidians exhibit a more extensive and variable group of sulfotransferase genes. Seven genes encoding proteins homologous to mammalian C4ST (C4ST‐like 1–7) and eight genes related to C6ST (C6ST‐like 1–8) were identified in *Ciona intestinalis* larvae. In the adult ascidians, only the C4ST‐like 2, 3, 5, and 7 and C6ST‐like 6 and 7 genes showed significant expression [23]. These animals belong to the phylum Chordata, subphylum Tunicata, and have been studied for their significant CNS regenerative capacity, even in adult individuals. Their CNS is composed of a brain, called the cerebral ganglion, which is divided into a cortex and a medulla, as well as a neural gland and other structures [24, 25]. GAGs have been purified and identified in ascidian viscera such as HS, HEP, CS, and DS [26, 27, 28, 29]. However, no specific GAGs have been identified in the CNS of ascidians, and their potential role in brain regeneration remains unknown. To investigate this, the brains of *Styela plicata* ascidians were chemically injured. Then, the composition of CS/DS disaccharides, enzyme gene expression, and phylogenetic analysis were examined throughout complete CNS recovery.

## Results

### Brain regeneration was observed 10 days after the neurotoxin injection

To evaluate neuroregeneration, the neurotoxin 3‐acetylpyridine (3‐AP) was used to cause death in high‐metabolic neurons [25]. Ultrastructural analysis (Fig. 1) revealed many neural and neuronal cells surrounded by an extracellular matrix composed of heterogeneous regions. One region mainly contained fibrillar material, and the other comprised a meshwork of axon terminals and dendrites along with the extracellular matrix. The neuronal soma presented a regular and preserved nuclear membrane containing euchromatin and heterochromatin, and the cytoplasm showed many preserved organelles (Fig. 1A). One day after the neurotoxin injection, the neuronal morphology changed. Fewer organelles were present, and many vacuoles were observed inside the cells, which is characteristic of vacuolar degeneration. Necroptotic neurons showing a swollen nucleus and a decrease in cell volume were observed. A glial cell containing many granules in its cytoplasm was observed extending a process to surround and engulf an apoptotic cell body. The fibrillar material and neuropile were indistinguishable in the extracellular matrix (Fig. 1B). Ten days after the 3‐AP injection, the tissue exhibited characteristics similar to those in the control group, without vacuoles, presenting cellular and nuclear membrane integrity, as well as distinct regions of the extracellular matrix containing fibrillar material and preserved neuropile (Fig. 1C).

**Fig. 1:**
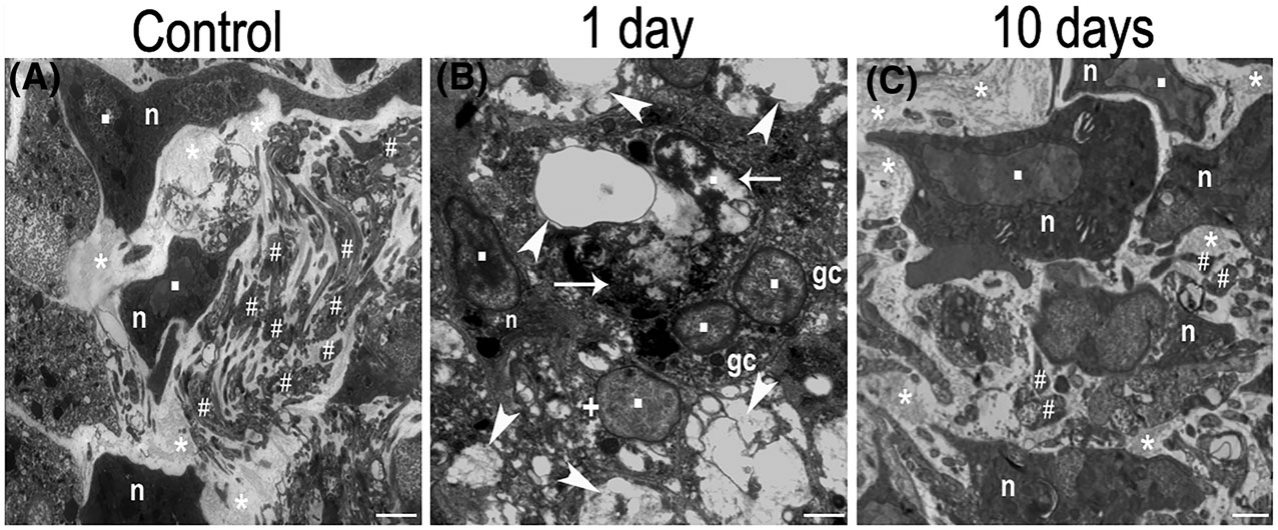
Ultrastructure of the cerebral ganglion during neuroregeneration. Ultrastructural analysis showed preserved neuronal morphology and an organized extracellular matrix in the control cerebral ganglion (A). 1 day after 3‐acetylpyridine (3‐AP) injection, neurons exhibited vacuolar degeneration, reduced organelles, necroptotic features, and glial cells engulfing apoptotic bodies, along with disruption of the extracellular matrix (B). 10 days after 3‐AP injection, tissue morphology was restored, resembling control conditions (C). *N* = 3 individuals per group. (*n*) neuron; (gc) glial cell; (+) necroptotic cell; (arrows) apoptotic body; (arrowheads) vacuoles; (square) nuclei; (*) fibrillar matrix; (#) neuropile. Scale: 2 μm.

Next, the histochemistry of the ascidian NC was performed (Fig. 2). The tissue sections were stained with hematoxylin and eosin (H&E) (Fig. 2A–D). The control group, which was only injected with the vehicle, showed preserved tissue architecture (Fig. 2A). One day after 3‐AP injection, vacuolization and intense cellular disorder were observed, especially in the cortical region (Fig. 2B, arrowheads). Five days postinjection, vacuolization was still observed in the cortex of the cerebral ganglion, albeit to a lesser extent (Fig. 2C). Ten days postinjection, the cerebral ganglion cortex showed neurons and glial cells; however, no vacuoles were observed, and the cytoarchitecture was indistinguishable from that of the control ganglion (Fig. 2D).

**Fig. 2:**
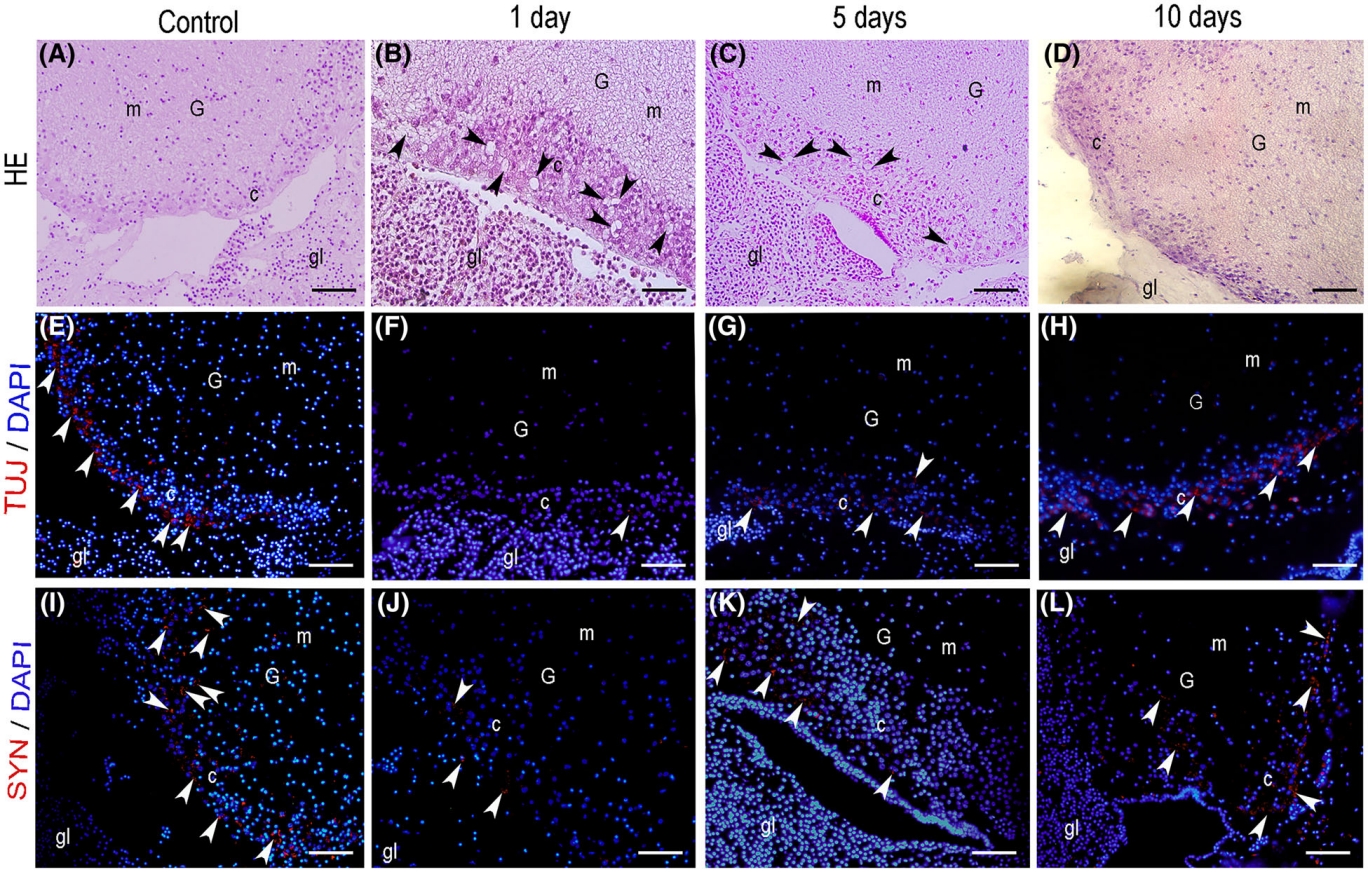
Histochemistry with hematoxylin and eosin and immunohistofluorescence of the neural complex (NC) of *Styela plicata* during neuroregeneration. Histochemistry with hematoxylin and eosin of the control NC, 1, 5, and 10 days after 3‐acetylpyridine (3‐AP) injection (A–D). Immunolocalization for β‐III‐tubulin (TUJ, red; E–H) and synaptophysin (SYN; red; I–L) in the control ganglion (E, I) and 1, 5, and 10 days after 3‐AP injection (F–H, J–L). 1 day after the neurotoxin application, neuronal disorder and vacuolation in the cortex were observed. TUJ and SYN were found in the cortical area of the cerebral ganglion except for 1 day after 3‐AP injection, when a reduction in labeling was observed. *N* = 4 individuals per group for routine staining; *N* = 3 individuals per group for immunohistofluorescence. DAPI (blue); (G) ganglion; (gl) neural gland; (C) cortex; (m) medulla; (black arrowheads) vacuoles; (white arrowheads) TUJ or SYN staining. Scale: 50 μm.

Immunofluorescence was used to evaluate aspects of neuroregeneration, such as neurogenesis and synaptogenesis. Labeling was performed for β‐III‐tubulin (TUJ) and synaptophysin (SYN), which are proteins that make up neuronal microtubules and presynaptic vesicles, respectively (Fig 2E–L). One day after neurotoxin injection, there was a decrease in labeling for TUJ and SYN compared to the control (Fig 2E–F, I–J). During the neuroregeneration process, a gradual increase in labeling was observed 5‐ and 10‐day postinjection, compared to 1 day after 3‐AP injection (Fig 2G–H, K–L).

The quantification of brain damage during regeneration was performed to confirm the image‐based findings (Fig. 3). Quantification of the vacuolated area of the cerebral ganglion showed a higher percentage of vacuolated area 1 day after neurotoxin injection compared to the control and 5‐ and 10‐day groups (Fig. 3A). Moreover, quantification of fluorescence per ganglion area showed decreases in TUJ and SYN labeling 1‐day postinjection compared to the control and 10‐day postinjection (Figs 3B,C). Ten days postinjection, the fluorescence per ganglion area was similar to the control. After confirming the temporal process of degeneration and regeneration through changes in morphology, as well as through staining of the cytoskeleton and synapses, the method was validated for investigating the involvement of GAGs during neuroregeneration.

**Fig. 3:**
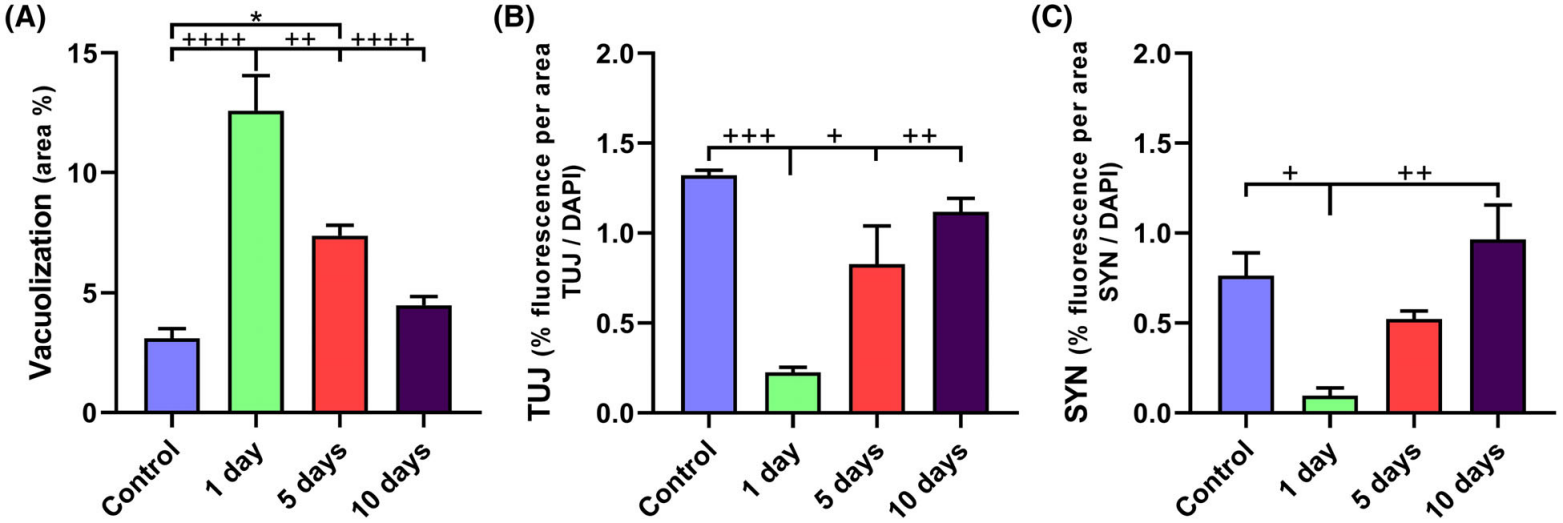
Quantifications of brain damage during regeneration. Quantification of the percentage of vacuolization per area of the cerebral ganglion stained with hematoxylin and eosin (A). Ratio of quantification of the percentage of β‐III‐tubulin (TUJ) (B) and synaptophysin (SYN) (C) fluorescences per fluorescent of DAPI area of the cerebral ganglion. 1 day after the 3‐acetylpyridine (3‐AP) injection, an increase in the vacuolated area was observed compared to the control and at 5 and 10 days. A reduction in TUJ and SYN staining was observed 1 and 5 days after 3‐AP injection compared to the control and at 10 days. *N* = 4 individuals per group for vacuolization analysis; *n* = 3 individuals per group for fluorescence quantification analysis. Normality was assessed using the Shapiro–Wilk test. Parametric data were analyzed using one‐way ANOVA followed by Tukey's multiple comparison *post hoc* test. (+) Comparison between the 1 day after 3‐AP injection group and the control and 10 days groups. (*) Comparison between the control and 1 and 5 days after 3‐AP injection groups. (#) Comparison between the 10 days after 3‐AP injection group and 1 and 5 days after 3‐AP injection groups. +,* *P* < 0.05; ++ *P* < 0.01; +++ *P* < 0.001; ++++ *P* < 0.0001.

### The expression of enzymes involved in the biosynthesis of chondroitin and dermatan sulfate changes during neuroregeneration

To evaluate the involvement of sulfation and epimerization enzymes in CS and DS biosynthesis during neuroregeneration, conventional and RT‐qPCR were performed. Conventional PCR demonstrated that C4ST‐L3, C4ST‐L5, C6ST‐L7, and UST are expressed in the adult brain, however, C6ST‐L1 is not expressed (Fig. S1). Analysis of the mRNA expression of the enzymes involved in CS and DS biosynthesis showed basal expression in the control NC and changes in expression at different stages of neuroregeneration (Fig. 4). DSE, an enzyme that catalyzes the epimerization of glucuronic acid into iduronic acid, exhibited high synthesis only 1 day after 3‐AP injection. After 5 and 10 days, DSE expression returned to normal ganglion levels (Fig. 4A). Subsequent analysis evaluated the expression of CST enzymes (C6ST‐L7, C4ST‐L3, C4ST‐L5, and UST), which promote sulfation at specific disaccharide positions. The data revealed an increase in C6ST‐L7, C4ST‐L3, and UST expression 1 day after 3‐AP injection (Fig 4B, C, and E), while no change in C4ST‐L5 expression was observed (Fig. 4D). Five and 10 days later, the mRNA levels of C6ST‐L7, C4ST‐L3, and UST returned to the levels found in the control group. Meanwhile, C4ST‐L5 mRNA levels increased 10 days after 3‐AP injection (Fig. 4D).

**Fig. 4:**
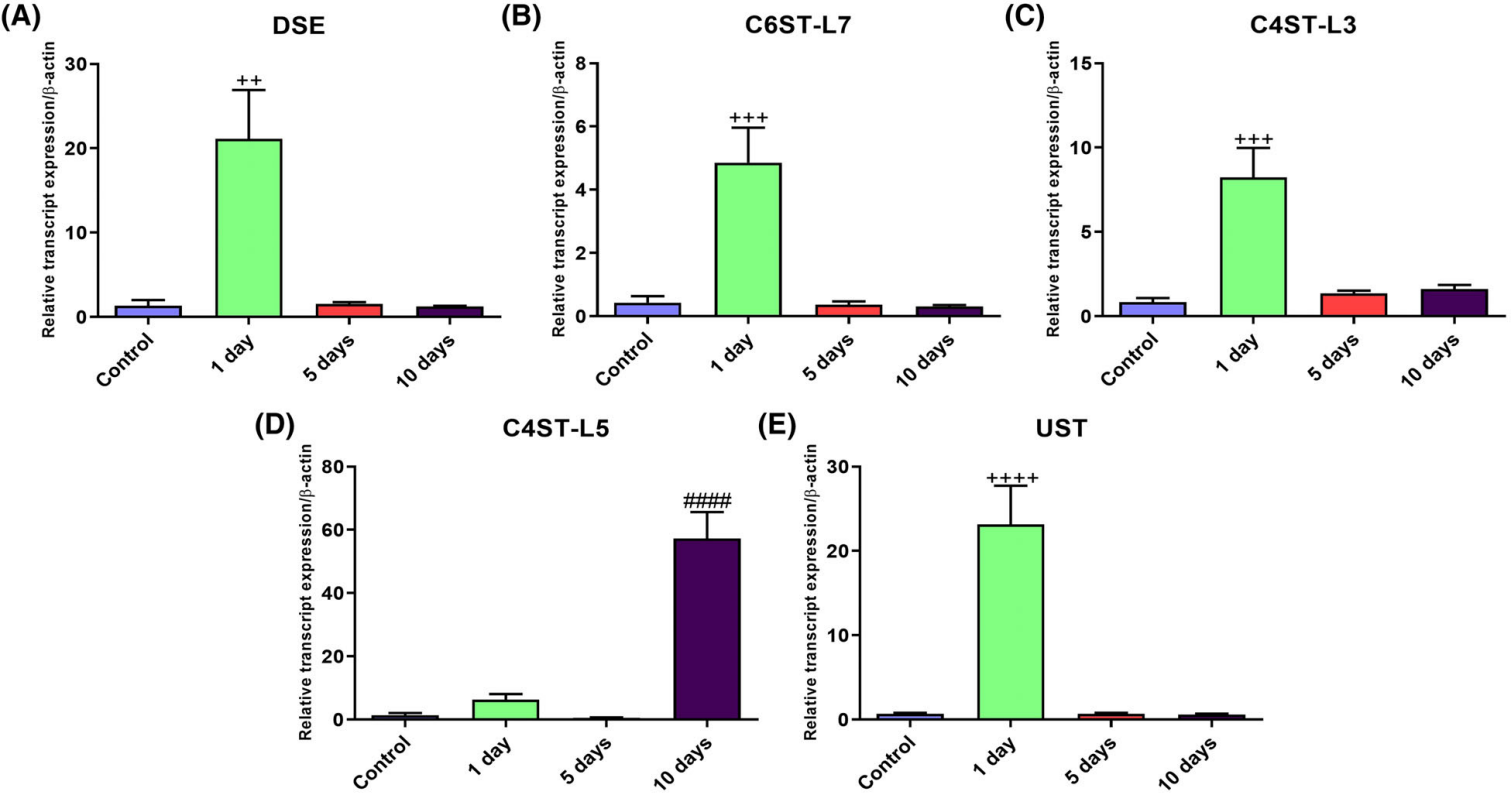
Quantitative analysis of real‐time gene expression of dermatan sulfate and chondroitin sulfate biosynthesis enzymes in the neural complex of *Styela plicata* during neuroregeneration. Expression of dermatan sulfate epimerase (DSE) (A), chondroitin 6‐O‐sulfotransferase‐like 7 (C6ST‐L7) (B), chondroitin 4‐O‐sulfotransferase‐like 3 (C4ST‐L3) (C), chondroitin 4‐O‐sulfotransferase‐like 5 (C4ST‐L5) (D), and 2‐O‐sulfotransferase (UST) (E) mRNA during neuroregeneration. The analysis showed basal mRNA expression levels in the control group and increased expression of DSE, C6ST‐L7, C4ST‐L3, and UST 1 day after 3‐acetylpyridine (3‐AP) injection. 5 and 10 days after 3‐AP injection, the levels of enzyme transcripts returned to basal levels. C4ST‐L5 was the only one that was overexpressed 10 days after 3‐AP injection. *N* = 4 pools per group (pool of four individuals). Normality was assessed using the Shapiro–Wilk test. Parametric data were analyzed using one‐way ANOVA followed by Tukey's multiple comparison *post hoc* test. (+) Comparison between the 1 day after 3‐AP injection group and the control and 5 and 10 days after 3‐AP injection groups. (#) Comparison between the 10 days after 3‐AP injection group and the control and 1‐ and 5 days after 3‐AP injection groups. ++ *P* < 0.01; +++ *P* < 0.001; ++++; #### *P* < 0.0001.

### Glycosaminoglycans are mostly found in the cortical region of the cerebral ganglion

After evaluating the expression of enzymes involved in the biosynthesis of C4S and D2,6S, immunolocalization of these GAGs was examined in the ascidian nervous tissue to determine their spatial distribution. Immunolocalization to identify C4S and D2,6S revealed labeling in the cortical region of the control ascidian brain at 1, 5, and 10 days (Fig. 5). C4S and D2,6S labeling was most prominent in the cortex of the cerebral ganglion, surrounding DAPI‐stained nuclei. Faint labeling was observed in the medulla, and some markings were present in cells between the gland lobules. One, 5, and 10 days after 3‐AP injection, the stained regions exhibited the same spatial distribution as the controls, and a quantitative analysis was performed using liquid chromatography methods.

**Fig. 5:**
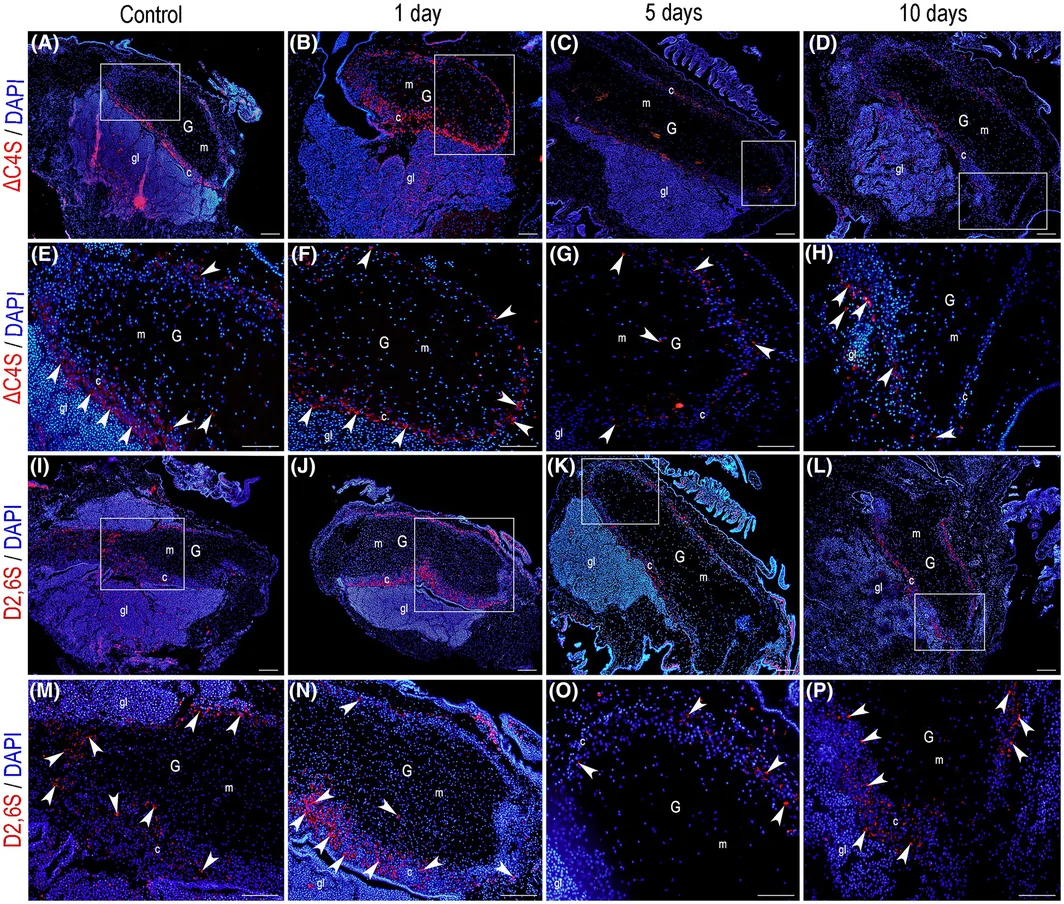
Immunohistofluorescence of chondroitin and dermatan sulfate in the neural complex of *Styela plicata* during neuroregeneration. Immunolocalization of chondroitin 4‐sulfate residues (∆C4S; red; A–H) and dermatan 2,6‐sulfate (D2,6S; red; I‐P) in the control ganglion (A, E, I, M) and 1, 5, and 10 days after 3‐acetylpyridine (3‐AP) injection (B–D, F–H, J–L, N–P). High‐magnification images of the cerebral ganglion show ∆C4S (E–H) and D2,6S (M–P) staining. ∆C4S and D2,6S staining were mainly found in the cortex of the cerebral ganglion in the glycocalyx or anchored to the cell membrane in all groups. *N* = 3 individuals per group. DAPI (blue); (G) ganglion; (gl) neural gland; (c) cortex; (m) medulla; (arrowheads) indicates immunofluorescence positive reaction; rectangle: area selected for high magnification. Scale: 50 μm.

### The production of chondroitin/dermatan 2,6 sulfate increases in the ascidian brain

To quantitatively determine which specific disaccharide units of CS and DS are present in the NC, a disaccharide analysis was carried out (Fig. 6). The disaccharides obtained from the NC of the ascidian during the different stages of neuroregeneration were compared to a molecular weight standard. Analysis of the graph showed that most of the peaks occurred at a retention time of approximately 18.8 min, which is characteristic of the ΔUA‐2SO4‐GalNAc‐6SO4 (∆Di2,6S) structure of D2,6S (Fig. 6A, a.2). The area under the curve indicated that over 90% of the disaccharides present in ascidian nervous tissue are ∆Di2,6S. Furthermore, analyzing the area under the curve for ∆Di2,6S across the different experimental groups revealed that the highest proportion of ∆Di2,6S was observed 5 days after 3‐AP injection (43.4%), followed by 1 day (24.1%), the control group (20.5%), and 10 days (12%) (Fig. 6B). Very small peaks characteristic of ∆UA‐GalNAc and ΔUA‐GalNAc‐4SO4 (∆Di4S) were detected during regeneration at retention times of approximately 10.5 and 14.4 min, respectively (Fig. 6A, a.1). This suggests a low concentration and limited involvement of the nonsulfated and C4S in the neuroregeneration process. However, the groups injected with 3‐AP exhibited the largest peaks of ∆Di4S 1 and 10 days after injection, respectively.

**Fig. 6:**
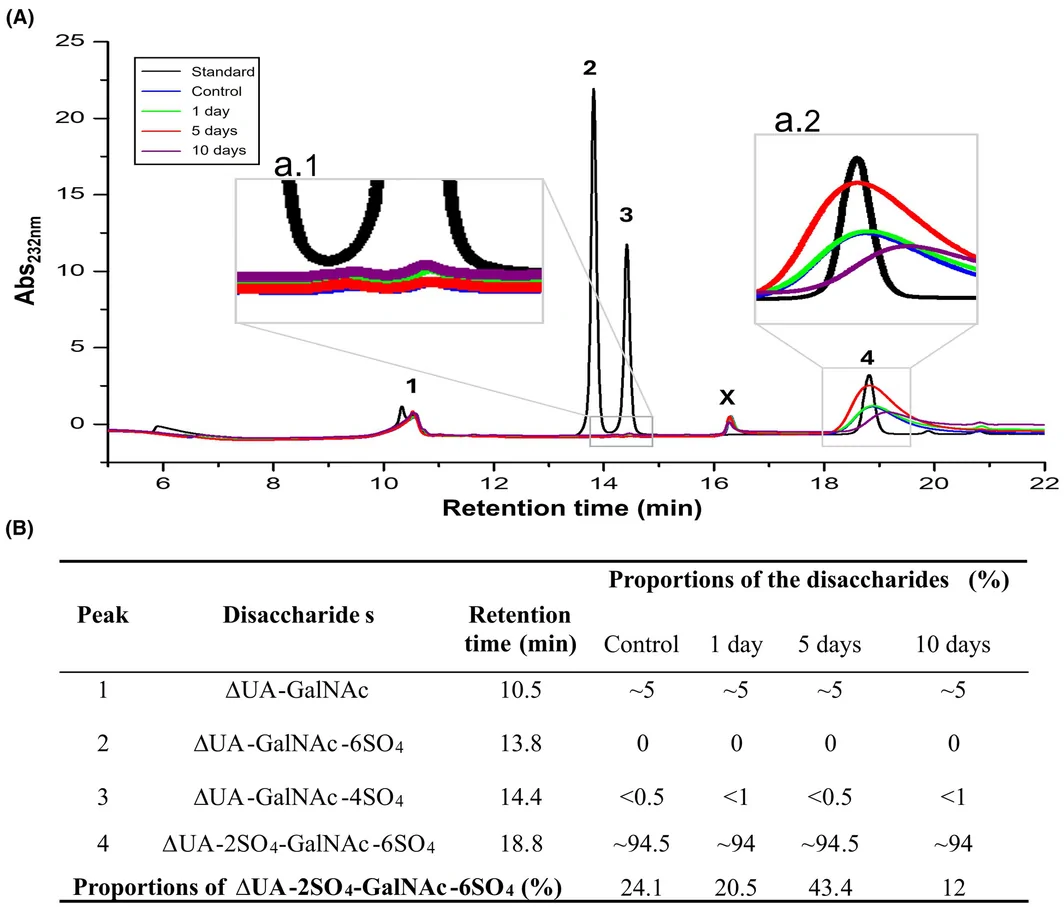
SAX‐HPLC separation and identification of disaccharides from the neural complex (NC) of *Styela plicata* during neuroregeneration. Peaks arising from treatment with chondroitin ABC lyase and chondroitin AC lyase (A); high magnification of peaks 3 and 4 (a.1, a.2) and proportion of disaccharides obtained by calculating the area under the curve (B). More than 90% of the disaccharides found in the ascidian NC were dermatan 2,6 sulfate (∆Di2,6S). The proportion of this disaccharide in the different stages of neuroregeneration revealed the highest amount 5 days after 3‐acetylpyridine (3‐AP) injection (red line), followed by 1 day (green line), the control group (blue line), and 10 days (purple line). *N* = 3 pools per group (pool of 10 individuals). Peak 1: ∆UA‐GalNAc; Peak 2: ∆UA‐GalNAc6SO4; Peak 3: ∆UA‐GalNAc4SO4; Peak 4: ΔUA‐2SO4‐GalNAc‐6SO4; X indicates an unidentified product.

### Dermatan 2,6 sulfate reverses impairment in siphon response time

Before the injections (ASW, 3‐AP, 3‐AP + D2,6S 1 μg/mL, and 3‐AP + D2,6S 5 μg/mL), all groups had similar times to siphon closure, and no statistical differences were observed. One day after 3‐AP injection, the siphon reflex slowed, presenting an increase in time to close the siphon compared to all groups at the same time (control, 3‐AP + D2,6S 1 μg/mL, and 3‐AP + D2,6S 5 μg/mL) and compared to day 0, before the injections, as well as compared to days 5 and 10 for all groups (control, 3‐AP + D2,6S 1 μg/mL, and 3‐AP + D2,6S 5 μg/mL), with statistical significance. Additionally, SST revealed that injecting 1 μg/mL D2,6S together with 3‐AP reduced the time it took for the ascidian siphons to close by 2 days compared to injecting only 3‐AP (Fig. 7).

**Fig. 7:**
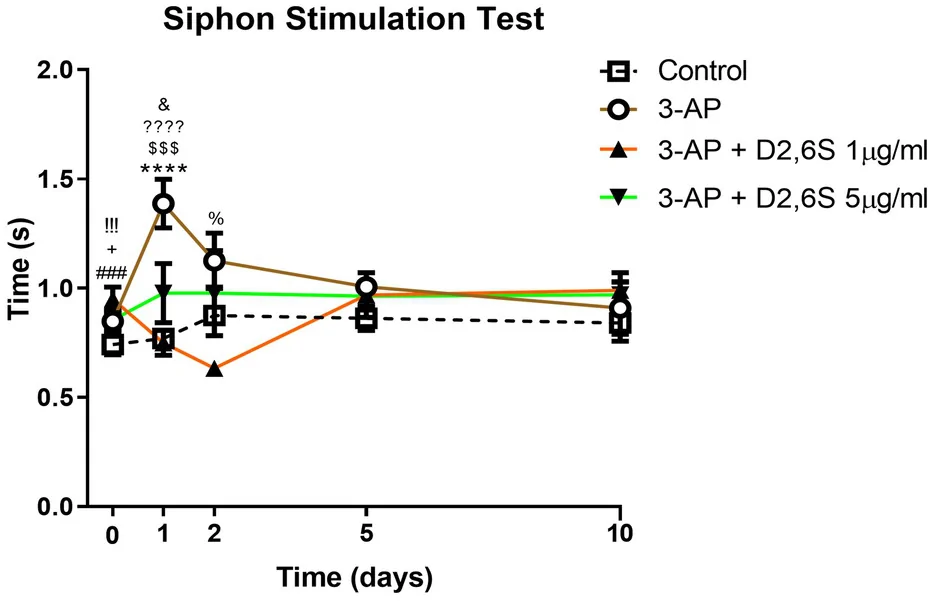
Siphon stimulation test after treatment with 3‐acetylpyridine (3‐AP) and dermatan 2,6 sulfate. Before injections, all groups showed similar siphon closure times. 1 day after 3‐AP injection, the siphon reflex was significantly slower, increasing closure time compared to all other groups and time points. Co‐injection of 1 μg/mL D2,6S with 3‐AP reduced closure time after 1 and 2 days compared to animals treated with 3‐AP alone at the same time points. *N* = 6 individuals per group. Normality was assessed using the Shapiro–Wilk test. Parametric data were analyzed using one‐way ANOVA followed by Tukey's multiple comparison *post hoc* test. (*) Differences between 3‐AP (day 1) versus (vs) control (days 0 and 1). ($) 3‐AP (day 1) vs control (day 10). (#) 3‐AP (day 0) vs 3‐AP (day 1). (?) 3‐AP (day 1) vs 3‐AP + D2,6S 1 μg/mL (days 1 and 2). (&) 3‐AP (day 1) vs 3‐AP + D2,6S 5 μg/mL (days 1, 2, 5, and 10). (%) 3‐AP (day 2) vs 3‐AP + D2,6S 1 μg/mL (day 2). (+) 3‐AP + D2,6S 1 μg/mL (day 0) vs 3‐AP (day 1). (!) 3‐AP + D2,6S 5 μg/mL (day 0) vs 3‐AP (day 1). (&; +; %) *P* ≤ 0.05; ($$$; ###; !!!) *P* ≤ 0.001; (****; ????) *P* ≤ 0.0001.

### Ascidian DSE is phylogenetically closer to vertebrate DSE 2

Considering that there are not many studies on ascidian DSE, including its phylogeny, unlike CST, the evolution of DSE among chordates, including cephalochordates, tunicates, and vertebrates, was evaluated. A search of the main genomes of the Ascidiacea class using human DSE 1 and DSE 2 sequences revealed two highly similar copies in *Ciona intestinalis* and three sequences in *Styela clava*. As previously reported, two types have been described in vertebrates: DSE 1 and DSE 2, which have been grouped into different clusters in the phylogeny (Fig. 8). The results showed that ascidian and amphioxus (*Branchiostoma belcheri*) sequences are closer to vertebrate DSE 2 sequences. In addition, analysis of the cluster formed by the class Ascidiacea showed that the *Ciona intestinalis* sequences formed a small cluster and that two of the three copies were found in *Styela clava*. The ‘DSE 2 *Styela clava a’* sequence grouped with the sequence found in the *Styela plicata* transcriptome, showing high similarity and a high confidence index (bootstrap = 100). Considering the ‘query cover’ and ‘percent identity’ homology indices obtained in BLAST^®^, the correspondence between the DSE 2 *Homo sapiens* sequence and the DSE 2 *Styela clava a* sequence resulted in a query cover of 94% and a percent identity of 35.85%. The DSE 2 *b* and *c* sequences resulted in query covers of 77% and 75%, respectively, and percent identities of 41.69% for both. The DSE 2 *Ciona intestinalis a* and *b* sequences resulted in query covers of 94% and 80%, and percent identities of 37.54% and 39.51%, respectively. Correspondence with the DSE 2 sequences from the *Styela plicata* transcriptome resulted in a query cover of 97% and a percent identity of 36.10% for the *Homo sapiens* DSE 2 sequence and query covers of 100% and 98% and percent identities of 68.14% and 44.57% for DSE 2 and both *Styela clava b* and *c* DSE 2 sequences. The query covers of *Styela plicata* and *Ciona intestinalis* DSE 2 *a* and *b* were 97% and 84% and the percent identities were 41.31% and 43.79%, respectively.

**Fig. 8:**
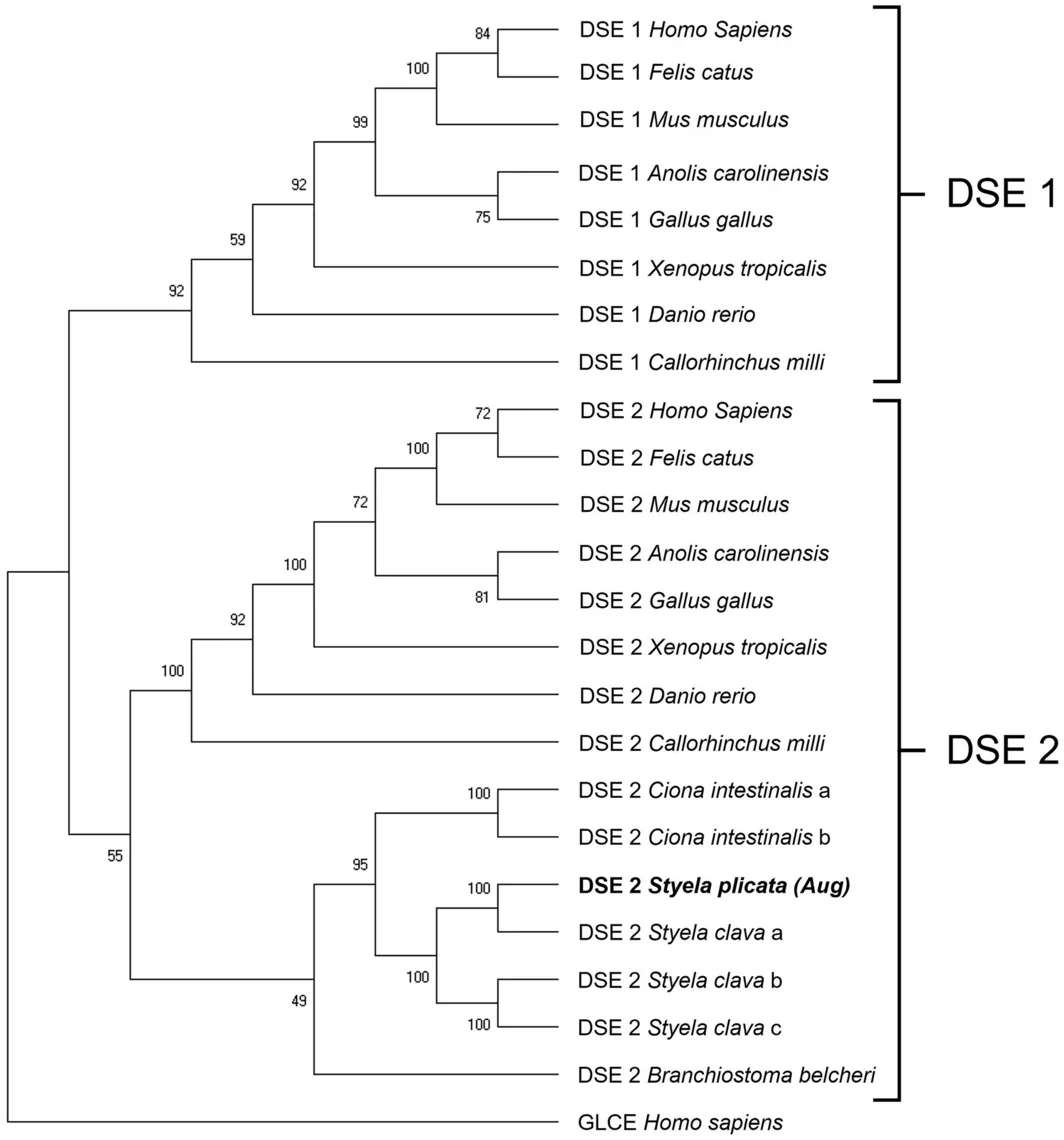
Phylogenetic analysis of dermatan sulfate epimerases type 1 (DSE 1) and type 2 (DSE 2) in organisms from phylum chordates and subphylum tunicates and cephalochordates. The ‘maximum likelihood’ method was used to describe the evolutionary relationships between the DSE 1 and DSE 2 sequences. Ascidians and cephalochordates have orthologs that are phylogenetically closer to vertebrate DSE 2 sequences. DSE 2 is the most common type found in the mammalian central nervous system. Bootstrap values (100 replications) are shown next to the tree branches. The D‐glucuronyl C5‐epimerase (GLCE) sequence was used as an outgroup. The (Aug) protein sequence was obtained through prediction performed by the AUGUSTUS software.

## Discussion

For the first time, this study described the dynamics of transcriptional levels and final disaccharide products of CS/DS during the degeneration and regeneration of the adult ascidian nervous system.

### Neurodegeneration and regeneration of the neural complex

Due to the limited regenerative capacity of the central nervous system (CNS) in most vertebrates [3], ascidians have emerged as powerful models for investigating the mechanisms of neurodegeneration and neuroregeneration. Since the nineteenth century and with growing interest over the last 15 years [24, 30], these tunicates have provided valuable insights into the biology of the chordate nervous system. In this study, we used the cosmopolitan ascidian *Styela plicata* and induced a chemical lesion through 3‐AP injection, a model previously validated by Medina *et al*. [25] and de Abreu *et al*. [31]. Medina *et al*. [25] documented cerebral ganglion vacuolization, reduced neuronal labeling, and caspase‐3 activation. They also performed transmission electron microscopy, confirming ultrastructural degeneration and apoptotic features in the injured CNS 1 day after injection. Here, we not only confirmed the degeneration markers and disruption of presynaptic connections during the acute phase, but also incorporated an intermediate time point (5‐day postinjection), at which morphological recovery becomes evident. Progressive restoration of synaptic labeling at 5 and 10 days is consistent with siphon functional recovery, as described by Medina *et al*. [25] and for the behavioral test performed here, as well as complete morphological regeneration, as observed here by ultrastructural analysis that showed extracellular matrix reorganization at 10‐day postinjection. The main focus of this study is the detailed characterization of CS and DS during the regenerative process.

### Regulation of chondroitin and dermatan sulfation

Considering that different types and subtypes of GAGs can regulate neurogenesis, neuritogenesis, and synaptic plasticity, the present study investigated CS and DS and their sulfation patterns [19, 32, 33]. The CS/DS biosynthetic enzymes expressed in the adult *Styela plicata* CNS are described here for the first time. However, Tetsukawa *et al*. [23] characterized the expression of several CSTs during different developmental phases and life stages in *Ciona intestinalis*. Their results showed that the C4ST‐L3, C4ST‐L5, C6ST‐L7, and UST isoforms are expressed in the adult stage of this species, a finding that was also observed in *Styela plicata* by our group. In addition, C6ST‐L1 was not reported in adult *Ciona intestinalis* [23] and, according to our analysis, it was also not found in the brain of adult *Styela plicata* during neuroregeneration.

The regulation of transcripts and final disaccharide products during regeneration was analyzed in this work for the first time. Although C4ST and C6ST transcripts increased after injury, disaccharide analysis revealed low levels of ΔDi4S disaccharide and high levels of ΔDi2,6S disaccharide in the ascidian CNS. Peak transcription of DSE, C6ST, and UST occurred at 1 day, preceding the peak of ΔDi2,6S disaccharide at 5 days. In contrast, although the magnitude of the ΔDi4S disaccharide peaks was moderate, the highest peaks were detected at 1‐ and 10‐day postinjection. These peaks were synchronous with the transcriptional peaks of C4ST‐L3 and C4ST‐L5, respectively. The discrepancy between the magnitude of the transcriptional peaks and final product disaccharide peaks suggests downregulation of C4ST‐L3 and C4ST‐L5 after transcription, resulting in enhanced ΔDi4S levels, though not to the same extent as the enhanced transcriptional levels of C4ST‐L3 and L5. Translational control mechanisms were reported to be influenced by cellular stress and neural dysfunction [34]. Considering that the degeneration and regeneration processes involve stress, this mechanism could be activated.

Comparative kinetic data on DS biosynthesis is scarce. Malmström *et al*. [17] demonstrated that D2,6S biosynthesis involves additional steps compared to C4S biosynthesis, including epimerization (DSE), 6‐O‐sulfation (C6ST), and subsequent 2‐O‐sulfation (UST), which is not yet fully elucidated [17, 35]. In our study, we observed the transcriptional levels of DSE, C6ST‐L7, and UST at 1 day, and we found the highest proportion of the final ΔDi2,6S disaccharide product at 5 days, suggesting that D2,6S may exhibit delayed biosynthetic kinetics compared to C4S, which requires only one sulfation step and does not require epimerization.

The contrast observed in the time period of the C4ST (L3 versus L5) isoform expression patterns (Fig 4C,D) suggests that the neuroregeneration process depends on different isoforms and that the production of these different enzymes may be related to the cell type producing the biosynthetic enzyme isoforms. Recently, our group detected the migration of PIWI‐positive cells (hemoblasts) to the CNS of the ascidian 1 day after the injection of 3‐AP, a marker of undifferentiated cells. Then, after 10 days, these cells started to express TUJ, a marker of young neurons [25, 31]. Therefore, perhaps the hemoblasts that migrated to the CNS produced the C4ST‐L3 isoform within 1 day and the differentiated cells produced the L5 isoform within 10 days.

Together, these findings suggest that ascidian neuroregeneration involves the coordinated transcriptional, kinetic, and possibly translational regulation of CS/DS biosynthesis, resulting in temporally controlled shifts in ECM sulfation patterns that culminate in structural regeneration.

### Comparison with vertebrate systems

Tunicates appear to have greater variability of CST enzymes than vertebrates. Eight C6ST (L1–L8) isoforms, seven C4ST (L1–L7) isoforms, and three UST (L1–L3) isoforms have been described in *Ciona intestinalis* [23]. In contrast, vertebrates have only two C6ST isoforms, three C4ST isoforms, and one UST isoform, respectively [17]. A recent genomic and evolutionary analysis of dermatan sulfate sulfotransferases in *Styela plicata* expanded this scenario by revealing an even broader repertoire, identifying 10 UST, nine C4ST, and five C6ST isoforms, which reinforces the remarkable expansion and diversification of these enzymes in ascidians [36]. This expanded enzymatic toolkit indicates a greater capacity for precise regulation of sulfation patterns in tunicates, potentially underlying the structural complexity and functional specialization of their GAGs.

Lin *et al*. [37] found that after cortical cerebral injury, mRNA levels of sulfation patterns on carbons 4 and 6 (C6ST‐1, C6ST‐2, and C4ST‐1) of galactosamine residue increased at the injury site in wild‐type mice compared to the uninjured side. Similar results were found in ascidians. After neural damage, the expression of both sulfotransferases (C6ST‐L7 and C4ST‐L3), which sulfate in different positions of chondroitin sulfate and dermatan sulfate, increased 1 day after 3‐AP injection. However, despite sulfation occurring on carbons 4 and 6 in ascidian nervous regeneration, the literature describes distinct roles for them. C6ST is responsible for sprouting axons and dendrites, while C4ST inhibits growth cones. This was observed in C6ST‐1 knockout mice, in which no significant regeneration occurred, unlike in wild‐type mice [37]. These findings highlight the importance of these enzymes and their respective products (6‐sulfated GAGs) for regeneration, as observed with the ascidian C6ST in the initial steps of neural renewal. On the other hand, enzymatic removal of CS chains using chondroitinase ABC has been shown to promote axon growth and generate more plastic tissue [38, 39]. Nevertheless, the 4‐O‐sulfation of CS has been associated with an inhibitory role in the regenerative process, based on studies that silenced C4ST in zebrafish and observed improvements in neuroregeneration [40].

In the adult mammalian CNS, recovery after injury is strongly limited by reduced ECM plasticity, marked by a predominance of C4S (approximately 90% of CS content) and the formation of C4S‐rich perineuronal nets and glial scars [38, 41, 42]. In contrast, ΔDi4S represents less than 1% of the disaccharides detected in the CNS of *Styela plicata*, even during the acute degeneration phase. Our group also reported low levels of GFAP+ astrocyte‐like cells at 1 and 10 days after 3‐AP injection [43]. Since these cells are central to C4S‐associated glial scar formation [38, 44], their absence may explain the limited CS production observed 1 day after 3‐AP exposure. Together with the ultrastructural data observed here, these findings suggest that no glial scar forms in this model, where vacuolization and neuronal loss are the main features of degeneration and may contribute to the complete reconstruction of the neural environment.

Our data demonstrate that D2,6S is abundantly present in the CNS of *Styela plicata*, as shown by immunofluorescence mainly in the cortical region of the cerebral ganglion, suggesting localization in the glycocalyx or attachment to the cell surface [22, 45]. During regeneration, there was a modest global increase of the total amount of D2,6S and C4S disaccharides without changing its regional localization. Although ΔDi2,6S accounts for ~ 90% of total GAGs in the viscera of *Phallusia nigra*, the viscera of *Styela plicata* predominantly contain 2,4‐sulfated disaccharides [26, 46]. Strikingly, our analysis revealed that over 90% of the disaccharides in the ascidian CNS correspond to ΔDi2,6S, indicating a tissue‐specific enrichment of this structurally complex and permissive GAG. In mammals, D2,6S is scarce in the adult CNS and is mainly detected in neurogenic niches or during the embryonic and neonatal stages, which are associated with higher plasticity [21, 47]. Experimental evidence further supports its pro‐regenerative role: DS promotes neuronal differentiation and neuritogenesis through IdoA‐dependent mechanisms [33], and D2,6S from *Phallusia nigra* exhibits greater neuritogenic and neuroprotective effects than D6S or C6S from mammals, highlighting the importance of IdoA epimerization and 2‐ and 6‐sulfation patterns [10]. Thus, the early upregulation of C6ST transcripts during ascidian regeneration may be linked to the differentiation of PIWI‐positive hemoblasts into neurons, as previously observed [31]. This process contributes to a permissive microenvironment for neural regeneration.

### Dermatan 2,6 sulfate as a neuroprotective molecule

The composition of DS extracted from *Phallusia nigra* exhibits significant bioactive properties related to neuroprotection and neuroregeneration. *In vitro* studies have demonstrated the potential of D2,6S to prevent rotenone‐mediated neuronal damage [8, 10]. This study demonstrates the neuroprotective effects of D2,6S extracted from *Phallusia nigra in vivo* for the first time. Our results showed that a 1 μg/mL dose of D2,6S was able to reverse and enhance the sensorimotor capacity of the ascidian 1–2 days after 3‐AP exposure, suggesting rapid functional recovery following neural injury. This effect may be associated with synaptic plasticity mechanisms, such as synaptogenesis or the strengthening of existing neuronal connections [32]. Treatment with 3‐AP alone induces a peak in siphon reflex time at 1‐day postexposure, which corroborates our observation of reduced synaptic labeling at the same time point and supports the occurrence of early synaptic impairment after neurotoxic injury.

Similarly, Nogueira *et al*. [48] reported motor impairment in *Styela plicata* after exposure to rotenone, evidenced by alterations in siphon movement, while treatment with L‐DOPA, which is commonly used in the palliative management of Parkinson's disease, partially restored this behavior. In this context, the effects observed for D2,6S may reflect its capacity to promote a more permissive extracellular environment for neural repair.

### Evolutionary aspects of DSE

Although the phylogeny of CSTs has been well explored [23, 36], the evolutionary background of DSE remains poorly understood. To our knowledge, this study is the first to investigate the evolutionary aspects of DSE in ascidians. Analysis of DS distribution in the animal kingdom revealed that mollusks, echinoderms, tunicates (ascidians), and vertebrates produce it. Based on the literature, DS is considered to have emerged more recently on the phylogenetic scale than other GAGs, such as CS and HS, which have been present since the beginning of evolution (except in Porifera) [28]. This could explain the limited variability of DSE isoforms in ascidians and their close resemblance to a single type of vertebrate DSE: DSE 2. This suggests that ascidian DSE is the precursor to the two types of DSE found in vertebrates. Interestingly, ascidian and vertebrate DSE show better homology with DSE 2, the most prevalent type in the vertebrate nervous tissue [49].

### Limitations and future directions

Although this study is relevant, some limitations should be considered. First, we did not directly manipulate the CS/DS biosynthetic enzymes, preventing us from demonstrating their causal role in regeneration. Further experiments are required to clarify the exact mechanisms activated during neuroregeneration.

## Conclusion

In summary, our data demonstrate that 3‐AP‐induced neurodegeneration in ascidians is followed by efficient regeneration that is associated with the dynamic remodeling of CS/DS sulfation patterns, particularly the enrichment of D2,6S. This study is the first to describe the presence of CS and DS in the CNS of adult ascidians and to characterize their modulation during neuroregeneration. Our findings suggest that specific DS motifs may contribute to the permissive regenerative environment observed in tunicates, offering evolutionary insights into CNS regeneration among chordates.

## Materials and methods

### Animal collection

A total of 233 adult *Styela plicata* ascidians were collected from Armação dos Búzios, Rio de Janeiro, Brazil, and maintained at 20 °C in an aerated aquarium with a controlled photoperiod (12 h of light and 12 h of darkness), pH (approximately 8.0), and salinity (34 PSU) for seven days prior to the experimental procedures. The animals were fed Artemia nauplii (~ 1.5 g/150 L), spirulina (~ 1.5 g/150 L), and Fuel™ (Aquavitro, Seachem, USA) [31]. The collection of the animals was authorized by Sisbio (No. 60330‐3).

### Experimental groups

The collected ascidians were divided into four experimental groups: Group 1 – Control (CON): Ascidians were injected with artificial seawater (ASW; [25]) and maintained in a separate aquarium; Group 2: Ascidians were injected with 65 mg/kg of 3‐AP and dissected and analyzed 1‐day postinjection; Group 3: Ascidians were dissected and analyzed 5‐day postinjection; Group 4: Ascidians were dissected and analyzed 10‐day postinjection (morphofunctional regeneration; [25]).

### Transmission electron microscopy

NCs from the control and experimental groups (1 and 10 days, *n* = 3 individuals per group) were dissected on ice and immediately fixed in 2.5% glutaraldehyde prepared in 0.1 m sodium cacodylate buffer in ASW (pH 7.6) for 2 h. The samples were then washed three times in ASW for 15 min each at room temperature. Postfixation was performed with 1% osmium tetroxide (Sigma, 75 633) for 30 min at room temperature. The tissues were then dehydrated in a graded acetone series and embedded in Epon 812 epoxy resin (Sigma/Fluka, Switzerland, 45 359). After resin polymerization, the blocks were sectioned using an RMC‐MT 600‐XL ultramicrotome (Leica). Ultrathin sections (60 nm) were mounted on 300 mesh copper grids, contrasted with 4% uranyl acetate for 20 min, followed by 1% lead citrate for 3 min, and examined with a JEOL JEM‐1011 transmission electron microscope operating at 80 kV. The microscope is available at the Rudolf Barth Electron Microscopy Platform at the Oswaldo Cruz Institute (Fiocruz).

### Routine histology

Ascidians (*n* = 4 per group) were anesthetized with 0.4 g/L menthol crystals, and the NCs of each animal were dissected. The NCs (both treated and control samples) were fixed in 4% paraformaldehyde diluted in ASW for 1 h, then dehydrated in ethanol, clarified in xylene, and embedded in Paraplast^®^ (Oxford Labware, St. Louis, MO). Five μm sections of the NCs were obtained using a rotary microtome (Leica RM 2245) and stained with hematoxylin and eosin (H&E). The morphology of the NCs was observed under an Olympus BX51 light microscope equipped with a DPF1 digital camera, and digital images (4080 × 3072 pixels) were captured. The neurotoxic effect was evaluated by quantifying vacuolization per area, which are white cavities, or vacuoles, considered a characteristic of brain degeneration. The area containing vacuoles was quantified in four sections taken from the most central part of the cerebral ganglion of four control and four experimental animals. The total area of each cerebral ganglion was considered to be 100%, and the apparent vacuoles were measured as a percentage of this total area using the program ImageJ 1.52. Bar graphs were constructed using GraphPad Prism version 8.00 from GraphPad Software (San Diego, CA) [25].

### Immunohistofluorescence

To determine the effects of 3‐AP and identify the types of glycosaminoglycans (GAGs) present in the NC, as well as their involvement in neuroregenerative processes, immunohistochemical reactions were carried out on three individuals per group. The fixation, dehydration, and embedding of the NC were performed according to the procedure described in routine histology. Subsequently, the sections were incubated in 100% methanol for 15 min to expose the epitopes. The sections were then immersed in phosphate‐buffered saline (PBS) solution (pH 7.4) made with ASW (PBS/ASW) and treated with 3% Triton X‐100 (Triton™ X‐100, Sigma‐Aldrich, CAS 9036‐19‐5) for 15 min. To block nonspecific sites, the tissue was incubated with 10% goat serum (Sigma‐Aldrich, G9023) for 1 h. Then, the sections were incubated overnight at 4 °C with the following primary antibodies, diluted in PBS/ASW: (1) Monoclonal anti‐β‐III‐tubulin (anti‐TUJ; Sigma, T2200) diluted 1:100, to identify young neurons; (2) Anti‐synaptophysin (anti‐SYN; Abcam, ab14692) diluted 1:100, to identify presynaptic vesicles; (3) Anti‐ΔC4S (Seikagaku, 270 432, 1:200 dilution) and anti‐D2,6S ([21]; 2A12, 1:100 dilution), produced from the DS of *Phallusia nigra*, which is 2,6 sulfated, to identify the GAG type. Next, the sections were incubated with a secondary antibody diluted in 10% goat serum (Alexa Fluor 555, Invitrogen, A21428, anti‐rabbit, 1:500 dilution) for SYN labeling or an Alexa Fluor 546 antibody (Invitrogen, A11030, anti‐mouse, 1:500 dilution) for labeling the other antibodies for 1 h. To stain the cell nuclei, the sections were exposed to 4′,6‐diamidino‐2‐phenylindole (DAPI; D8417). For negative controls, the primary antibodies were omitted from the incubation steps (Fig. S2). To confirm the specificity of the ΔC4S antibody staining, NC sections were pretreated with chondroitinase AC (2 mIU/section) at 37 °C in a humid chamber, then processed for immunohistofluorescence as described above [21]. The images were captured using the previously described Olympus microscope. Immunofluorescence for TUJ and SYN was analyzed using the ImageJ 1.52a software to quantify the fluorescent area percentage of the brain. The analysis was performed using three images from three animals in each group (control, 1 day, 5 days, 10 days) [25]. The ratio of TUJ or SYN fluorescence per DAPI‐fluorescent nuclei was expressed as a percentage in the graphs.

### Real‐time qPCR

RNA was extracted from the control and experimental groups (*n* = 4 pools per group; pool of four individuals) using TRIzol™ (Invitrogen, 15 596 026) and Direct‐zol™ RNA MiniPrep (Zymo Research, R2050), according to the manufacturers' instructions. Then, complementary DNA (cDNA) was synthesized from the RNA using a High‐Capacity cDNA Reverse Transcription Kit (Thermo Fisher Scientific, 4 368 814) according to the manufacturer's instructions. Qualitative PCR with 3% agarose electrophoresis was previously carried out to select the main genes expressed by the adult brain of ascidians. A quantity of 1.19 μg of cDNA was used as a template for RT‐qPCR analysis with SYBR Green‐based detection. RT‐qPCR reactions were carried out in duplicate (technical replicates), and melting curves were examined to ensure single products. The results were quantified using the ‘delta–delta Ct’ method and normalized to β‐actin transcript levels, as previously described [43, 48]. The primer sequences of C6ST‐like 1 (C6ST‐L1), C6ST‐like 7 (C6ST‐L7), C4ST‐like 3 (C4ST‐L3), C4ST‐like 5 (C4ST‐L5), DSE, and UST, as well as the GenBank accession numbers of the sequences analyzed, are provided as (Table S1). These sequences were chosen because previous research identified expression of these genes in the adult stages of *Ciona intestinalis*. Specifically, C6ST‐L1 was found only in the neural structures of tadpoles and was chosen as the negative expression control.

### Glycosaminoglycans purification and disaccharide analysis

The protocol was carried out according to the methods established by de Sousa *et al*. [8]. The NCs were dissected into (*n* = 3 pools per group; pool of 10 individuals) and underwent delipidation in acetone for 24 h at 4 °C, followed by protein digestion in papain diluted in digestion buffer (0.1 m sodium acetate buffer at pH 5.5, 5 mm EDTA, and 5 mm cysteine) at 60 °C. Then, the total polysaccharides were precipitated in 100% ethanol. Quantification was performed by colorimetric assay using the carbazole method [50]. About 0.5 mg/mL of glucuronolactone (Sigma; 32 449–92‐6) was used as the standard. The final reaction was read in a Shimadzu UVmini‐1240 spectrophotometer at 525 nm.

To obtain the CS and DS disaccharides, 54,5 μg of polysaccharides were subjected to exhaustive enzymatic digestion with approximately 0.05 U each of chondroitin ABC lyase (Chase ABC; Sigma, C3667, USA) and chondroitin AC lyase (Chase AC; Sigma, C2262, USA) in sodium acetate buffer (0.05 m, pH 7.0) at 37 °C for 5 days, with the following additions: on days 1, 3, and 5, 0.5 μL of Chase ABC was added; on days 2 and 4, 0.7 μL of Chase AC was added. The disaccharides obtained from the NCs, along with commercial CS and DS disaccharide standards, were applied to a Supelcosil SAX1 column (5 μm, 250 × 4.6 mm; Sigma, 59 138, USA) connected to an HPLC system (Shimadzu, Kyoto, Japan). The disaccharides were eluted using a linear gradient until 1 m NaCl (pH 3.5) at a flow rate of 1 mL·min ^−1^. The SAX1 eluent was continuously monitored at 232 nm [10]. The proportion of disaccharides was calculated by integrating the chromatographic peaks to determine the area under the curve (AUC). Peak integration and data processing were performed using Origin 8.5 software (OriginLab, Northampton, MA, USA), and the results were expressed as a percentage.

### Behavioral test

Ascidians (n = 6 individuals per group) were mechanically stimulated in the oral siphon before (day 0) and daily after (days 1, 2, 5, and 10) the administration of the 3‐AP neurotoxin [24, 25, 48, 51]. Another group of animals (control) was injected only with the vehicle for the neurotoxin (ASW), while two other groups were injected with 3‐AP and two concentrations (1 and 5 μg/mL) of D2,6S, which was extracted and purified from the ascidian *Phallusia nigra* [10]. The response (closure of the siphons) was recorded using a Panasonic Lumix DMC‐FZ38 camera, and the time it took the animals to close their siphon was measured using Clipchamp: Video Editor & Maker (Microsoft, USA).

### Phylogenetic analysis

The DSE 1 and DSE 2 protein sequences were searched in representative species of all major chordate groups, including tunicates and cephalochordates, in the NCBI database (https://www.ncbi.nlm.nih.gov/pubmed/). Tunicate genomes of *Ciona intestinalis* [52] and *Styela clava* [53] and the *Styela plicata* transcriptome were searched using BLAST [54]. Then, AUGUSTUS [55] was used to obtain the respective protein sequences. Amino acid sequences were aligned using MUSCLE [56]. Gaps were manually removed, and the phylogenetic analysis was performed using MEGA version X [57]. The best amino acid substitution model was JTT + G, and a maximum likelihood analysis was performed in MEGA version X with 100 bootstrap replicates. The GenBank accession numbers of the analyzed sequences are provided in the (Table S1).

### Statistical analysis

As explained above, raw data were collected for each experiment. The Shapiro–Wilk test was applied to check for normality. Parametric data were analyzed using one‐way ANOVA, followed by Tukey's multiple comparison *post hoc* test. The results were expressed as the mean ± standard error of the mean, and differences were considered significant at *P* < 0.05. All analyses were conducted using GraphPad Prism version 8.01.

## Author contributions

CMB, conceptualization, funding acquisition, supervision, writing – original draft, review and editing; RNF, methodology, investigation, review, and editing; MSGP, methodology, investigation, review, and editing; BCR, methodology, investigation, and review; SOP, methodology, investigation, review, and editing; TMP, conceptualization, data curation, formal analysis, writing – original draft, review, and editing.

## Conflict of interest

The authors declare no conflicts of interest.

## Supporting information


**Fig. S1.** Qualitative PCR detects presence of chondroitin sulfotransferases in the neural complex (NC) of Styela plicata.
**Fig. S2.** Negative controls of immunohistofluorescence of the neural complex (NC) of *Styela plicata* during neuroregeneration.
**Table S1.** Accession codes of dermatan sulfate epimerase 1 and type 2 (DSE 1 and DSE 2) and GLCE (outgroup) sequences and primers used in this study
**Table S2.** Data availability‐ Portal *et al*., 2026.

## Data Availability

The raw data are shown as Table S2.
