# Supplementary material for: Characterization of dermatan sulfate and chondroitin sulfate profiles during brain regeneration in Styela plicata
Source: FEBS J. 2026 May 21;293(21):6569–85. doi: 10.1111/febs.70598 (PMC13731937; doi:10.1111/febs.70598)
Supplement: Supplementary file 1 [file FEBS-293-6569-s001.pdf]

Supplementary Figure 1. Qualitative PCR detects presence of chondroitin sulfotransferases in the neural complex (NC) of *Styela plicata*.

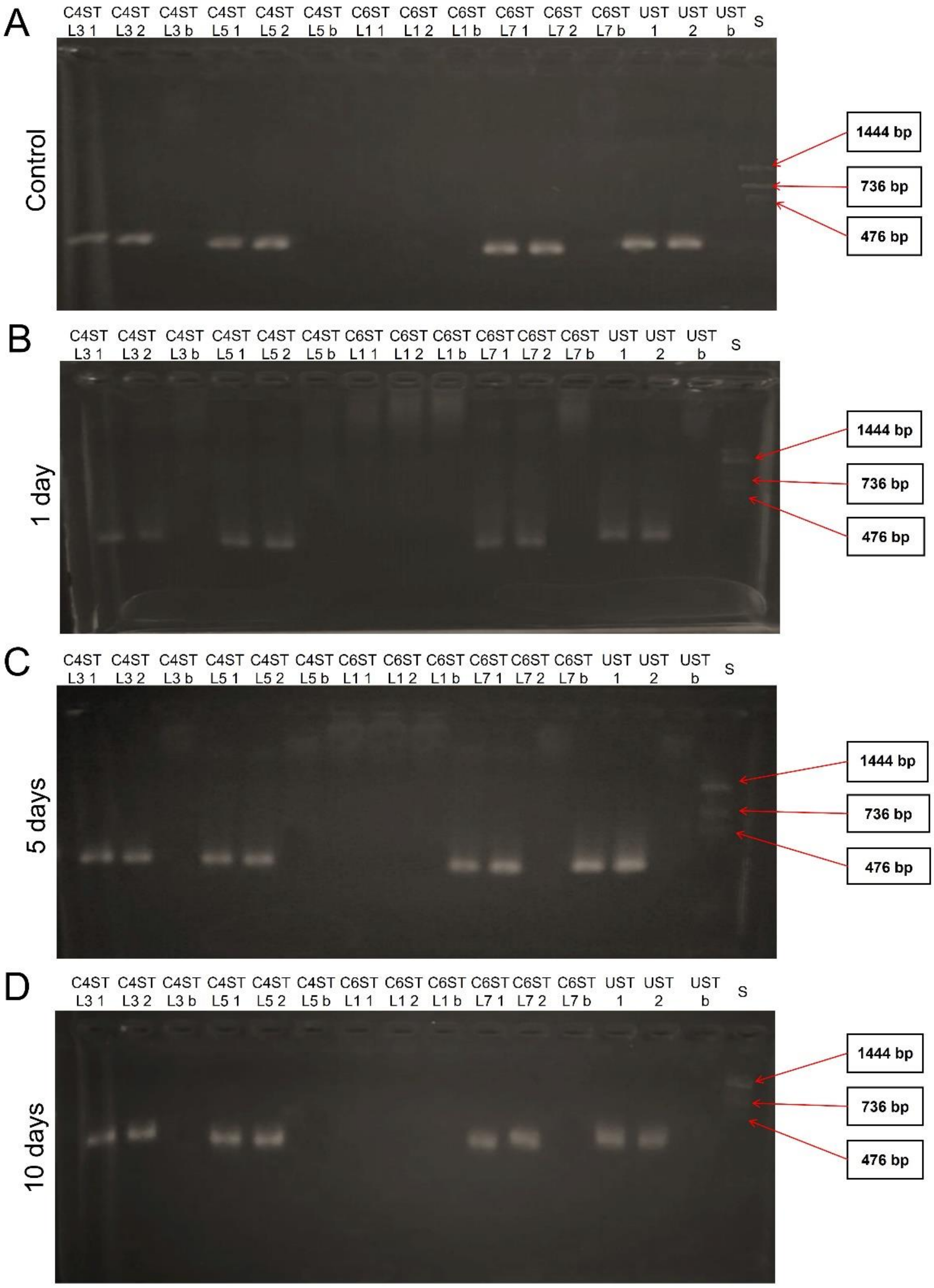

Amplification of the genes C4ST-L3, C4ST-L5, C6ST-L1, C6ST-L7 and UST is shown. Control ganglion (A) 1, 5 and 10 days after 3-acetylpyridine injection (B-D). Amplicon sizes ranged from 90 to 130 base pairs (bp). Expression of the target genes was detected in control NCs and across different time points of neuroregeneration, except for C6ST-L1, which was used as a negative expression control. (b) Blank reactions (no-template control); (S) molecular weight marker.

Supplementary Figure 2. Negative controls of immunohistofluorescence of the neural complex (NC) of *Styela plicata* during neuroregeneration.

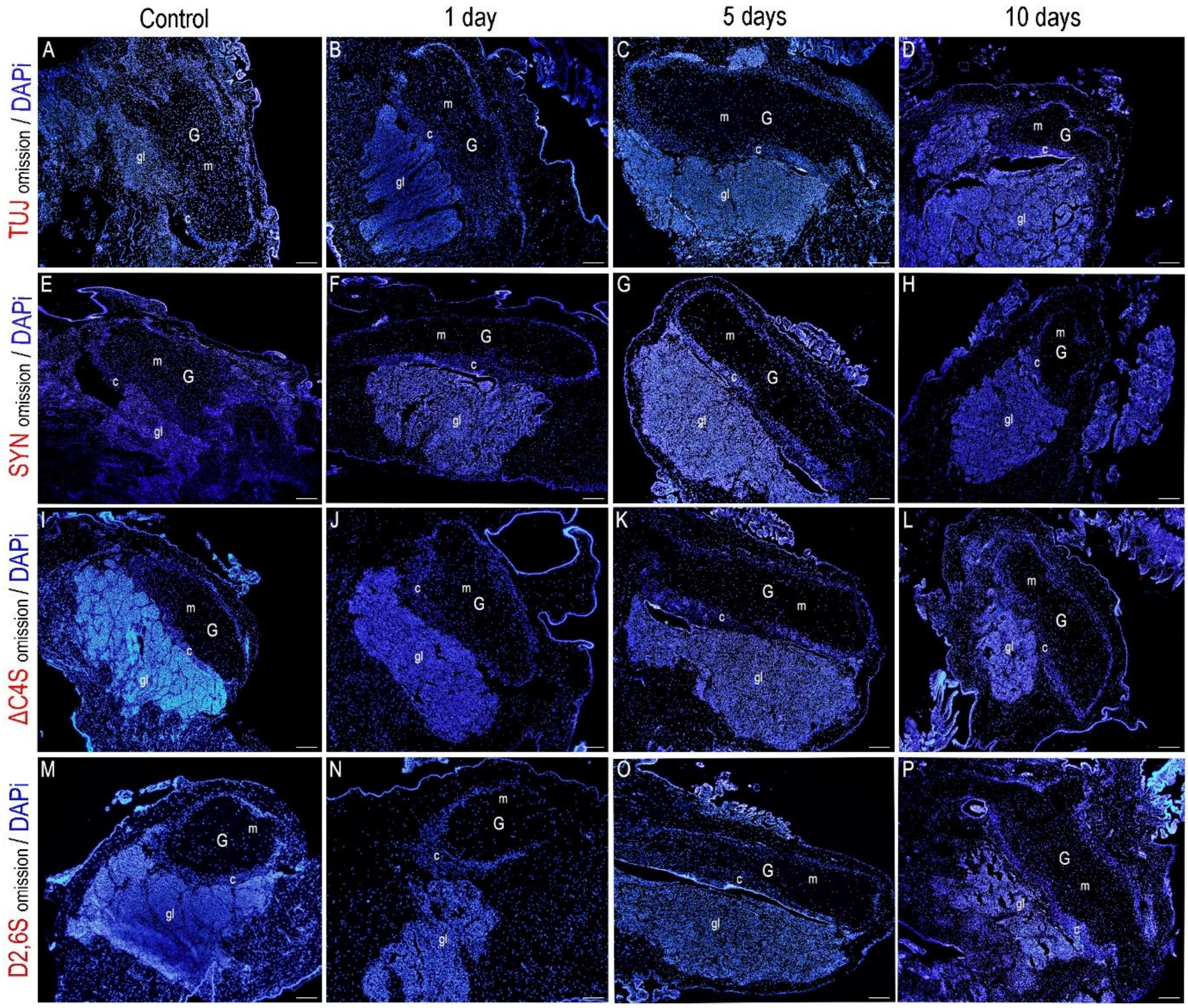

Omission of anti-β-III-tubulin (TUJ; A-D), anti-synaptophysin (SYN; E-H), anti-chondroitin 4-sulfate residues (ΔC4S; I-L) and anti-dermatan 2,6-sulfate (D2,6S; M-P) antibodies from the control ganglion (A, E, I, M), 1, 5 and 10 days after injection of 3-acetylpyridine (B-D, F-H, J-L, N-P, respectively). With the omission of primary antibodies, it is not possible to observe the red markings, proving the efficiency of the immunostaining in Figures 2 and 5. DAPI (blue), (G) Ganglion, (gl) neural gland, (c) cortex, (m) medulla. Scale: 50 μm.

Supplementary Table S1. Accession codes of dermatan sulfate epimerase 1 and type 2 (DSE 1 and DSE 2) and GLCE (outgroup) sequences and primers used in this study.

Specie	GENBANK CODE DSE 1	GENBANK BASE
Homo sapiens	NP_001074445.1	NCBI
Mus musculus	NP_766096.1	NCBI
Felis catus	XP_019686664.2	NCBI
Anolis carolinenses	XP_016853226.1	NCBI
Gallus gallus	XP_419777.3	NCBI
Xenopus tropicalis	NP_001120268.1	NCBI
Callorhinchus milii	XP_007897828.1	NCBI
Danio rerio	NP_001025396.1	NCBI
Ciona intestinalis a	XXXX	XXXX
Ciona intestinalis b	XXXX	XXXX
Styela clava a	XXXX	XXXX
Styela clava b	XXXX	XXXX
Styela clava c	XXXX	XXXX
*Styela plicata**	XXXX	XXXX
Branchiostoma belcheri	XXXX	XXXX

Specie	GENBANK CODE DSE 2	GENBANK BASE
Homo sapiens	NP_115536.2	NCBI
Mus musculus	NP_001074785.1	NCBI
Felis catus	XP_019670965.1	NCBI
Anolis carolinenses	XP_003219769.2	NCBI
Gallus gallus	XP_004935280.1	NCBI
Xenopus tropicalis	XP_002939660.2	NCBI
Callorhinchus milii	XP_007895427.1	NCBI
Danio rerio	XP_696502.2	NCBI
Ciona intestinalis a	XP_002130087.1	NCBI
Ciona intestinalis b	XP_026689514.1	NCBI
Styela clava a	XP_039248381.1	NCBI
Styela clava b	XP_039249456.1	NCBI
Styela clava c	XP_039249458.1	NCBI
*Styela plicata**	*	*
Branchiostoma belcheri	XP_019628749.1	NCBI

*Nucleotide sequence derived from transcriptome. Protein sequence predicted by AUGUSTUS software.

Specie	GENBANK CODE GLCE	GENBANK BASE
Homo sapiens	NP_001311020.1	NCBI

PRIMERS

DSE_FWD	TCCGTTGGACGAAGTCGATG
DSE_REV	ACAATTAGGCGAGTGCGACA
C6ST-like 7_FWD	TCGGTTGCTTACGAGCACTT
C6ST-like 7_REV	AGGGTGTAGCAACCAGGGTA
C4ST-like 3_FWD	TTGGAAGTACAAGCCGCACT
C4ST-like 3_REV	CCCGATCCCTCACTTTTGGT
C4ST-like 5_FWD	CGACTGAAGGGCTCTCGAAC
C4ST-like 5_REV	CAACGACAATCGCACACAGG
UST_FWD	CCACCACCTCCTCGTTGATT
UST_REV	CGATTTGGTGAGAAACAGCCA
Act_FWD	GATTCTGGCGATGGCGTTTC
Act_REV	GTCAGATCACGACCAGCCAA

Supplementary Table S2. Data availability- Portal et al., 2026

Figure 3A - Vacuolization	
Experimental group	area %
Control n=1	2.557
Control n=2	4.212
Control n=3	2.381
Control n=4	3.236
1 day n=1	16.332
1 day n=2	9.228
1 day n=3	11.942
1 day n=4	12.879
5 days n=1	7.033
5 days n=2	6.804
5 days n=3	8.663
5 days n=4	6.998
10 days n=1	5.054
10 days n=2	3.449
10 days n=3	4.866
10 days n=4	4.553

Figure 3B - TUJ fluorescence	
Experimental group	area %
Control n=1	1.376
Control n=2	1.294
Control n=3	1.291
1 day n=1	0.264693
1 day n=2	0.170359
1 day n=3	0.241597
5 days n=1	0.408029
5 days n=2	1.102
5 days n=3	0.966667
10 days n=1	1.209
10 days n=2	0.970664
10 days n=3	1.173

Figure 3C - SYN fluorescence	
Experimental group	area %
Control n=1	0.923913
Control n=2	0.518715
Control n=3	0.849744
1 day n=1	0.041561
1 day n=2	0.063706
1 day n=3	0.180549
5 days n=1	0.447557
5 days n=2	0.605161
5 days n=3	0.515101
10 days n=1	0.659663
10 days n=2	1.317
10 days n=3	0.914286

Figure 4 - RT-qPCR

Figure 4A - DSE	
Experimental group	Value
Control n=1	3.254
Control n=2	0.153778
Control n=3	1
Control n=4	0.965427
1 day n=1	37.029
1 day n=2	10.025
1 day n=3	16.019
1 day n=4	21.377
5 days n=1	1.040
5 days n=2	1.972
5 days n=3	1.669
5 days n=4	1.613
10 days n=1	1.319
10 days n=2	1.375
10 days n=3	1.112
10 days n=4	1.278

Figure 4B - C6ST-L7	
Experimental group	Value
Control n=1	1
Control n=2	0.050996
Control n=3	0.420472
Control n=4	0.242506
1 day n=1	3.198
1 day n=2	3.237
1 day n=3	5.112
1 day n=4	7.881
5 days n=1	0.0949
5 days n=2	0.299726
5 days n=3	0.554339
5 days n=4	0.516145
10 days n=1	0.18872
10 days n=2	0.360429
10 days n=3	0.304706
10 days n=4	0.38871

Figure 4C - C4ST-L3	
Experimental group	Value
Control n=1	1
Control n=2	0.212098
Control n=3	1.314
Control n=4	0.862972
1 day n=1	6.386
1 day n=2	4.284
1 day n=3	11.752
1 day n=4	10.506
5 days n=1	1.105
5 days n=2	1.619
5 days n=3	1.669
5 days n=4	1.040
10 days n=1	1.159
10 days n=2	1.438
10 days n=3	1.623
10 days n=4	2.257

Figure 4D - C4ST-L5	
Experimental group	Value
Control n=1	3.528

Figure 4E - UST	
Experimental group	Value
Control n=1	0.677071

Control n=2	1	Control n=2	0.722206
Control n=3	0.589835	Control n=3	1
Control n=4	0.323601	Control n=4	0.31868
1 day n=1	3.207	1 day n=1	19.231
1 day n=2	4.947	1 day n=2	34.537
1 day n=3	5.427	1 day n=3	13.047
1 day n=4	11.581	1 day n=4	25.725
5 days n=1	0.135742	5 days n=1	0.423639
5 days n=2	0.875238	5 days n=2	0.992159
5 days n=3	0.552257	5 days n=3	0.771192
5 days n=4	0.594847	5 days n=4	0.547175
10 days n=1	79.606	10 days n=1	0.360895
10 days n=2	55.677	10 days n=2	0.412599
10 days n=3	54.786	10 days n=3	0.870658
10 days n=4	38.882	10 days n=4	0.724786

Figure 7 - Siphon Stimulation Test

Time (days)	Control (seconds)					
	n=1	n=2	n=3	n=4	n=5	n=6
0	0.81	0.7	0.56	0.72	0.9	0.76
1	0.84	0.72	0.67	0.8	0.89	0.7
2	0.94	0.97	0.73	0.89	0.91	0.81
5	0.86	0.87	0.71	1.04	0.79	0.9
10	1.06	1.01	0.76	0.6	0.78	1

Time (days)	3-AP (seconds)					
	n=1	n=2	n=3	n=4	n=5	n=6
0	0.97	0.88	0.72	0.87	0.9	0.75
1	1.35	0.97	1.69	1.28	1.69	1.34
2	1.69	1.26	1	1.04	0.93	0.83
5	0.97	1.07	1	1.07	0.98	0.95
10	1.41	0.96	0.53	0.76	0.98	0.81

Time (days)	3-AP + D2.6S 1μg/ml (seconds)					
	n=1	n=2	n=3	n=4	n=5	n=6
0	0.93	0.97	0.79	0.88	0.93	1.2
1	0.68	0.75	0.55	0.74	0.8	0.98
2	0.59	0.57	0.57	0.66	0.68	0.73
5	0.77	1.05	1.04	0.77	0.78	1.4
10	0.9	1.23	1.18	0.68	1	0.95

Time (days)	3-AP + D2.6S 5μg/ml (seconds)					
	n=1	n=2	n=3	n=4	n=5	n=6
0	0.73	0.95	0.75	0.86	1.02	0.82
1	0.61	0.87	1.02	0.67	1.49	1.2
2	0.62	0.5	0.87	1.02	1.86	0.99
5	0.77	0.91	1.01	1.03	0.92	1.14
10	1.09	0.91	1.02	1.01	0.77	1.07
